# Infrared probe of pseudogap in electron-doped Sr_2_IrO_4_

**DOI:** 10.1038/s41598-017-10725-z

**Published:** 2017-09-05

**Authors:** J. H. Seo, G. H. Ahn, S. J. Song, X. Chen, S. D. Wilson, S. J. Moon

**Affiliations:** 1 Department of Physics, Hanyang University, Seoul, 04763 Korea; 2 Department of Physics, Boston College, Chestnut Hill, Massachusetts, 02367 USA; 3 Materials Department, University of California, Santa Barbara, California, 93106 USA

## Abstract

We report on infrared spectroscopy experiments on the electronic response in (Sr_1−*x*_La_*x*_)_2_IrO_4_ (*x* = 0, 0.021, and 0.067). Our data show that electron doping induced by La substitution leads to an insulator-to-metal transition. The evolution of the electronic structure across the transition reveals the robustness of the strong electronic correlations against the electron doping. The conductivity data of the metallic compound show the signature of the pseudogap that bears close similarity to the analogous studies of the pseudogap in the underdoped cuprates. While the low energy conductivity of the metallic compound is barely frequency dependent, the formation of the pseudogap is revealed by the gradual suppression of the featureless conductivity below a threshold frequency of about 17 meV. The threshold structure develops below about 100 K which is in the vicinity of the onset of the short-range antiferromagnetic order. Our results demonstrate that the electronic correlations play a crucial role in the anomalous charge dynamics in the (Sr_1−*x*_La_*x*_)_2_IrO_4_ system.

## Introduction

The layered perovskite variant iridate Sr_2_IrO_4_ has attracted considerable attentions as a candidate correlated electron material from which unconventional superconductivity is predicted to emerge when external carriers are introduced. Sr_2_IrO_4_ bears close similarities to the parent compounds of high-temperature cuprate superconductors. The electromagnetic state of Sr_2_IrO_4_ is described by a pseudospin 1/2 antiferromagnetic Mott state where the cooperation between the electronic correlations and the spin-orbit coupling leads to the formation of the effective total angular momentum *J*_eff_ = 1/2 Mott insulating state^1, 2^. The magnetic excitation spectra of Sr_2_IrO_4_ is well described by antiferromagnetic Heisenberg model with a pseudospin 1/2 on a quasi-two-dimensional square lattice^3, 4^. Given these similarities, theoretical studies suggest that a singlet *d*-wave or a triplet *p*-wave high-temperature superconductivity could emerge in electron or hole-doped Sr_2_IrO_4_, respectively^5–7^. Subsequent experimental effort searching for high-temperature superconductivity indeed uncovered the parallel phenomenology to the cuprates including the *d*-wave gap^8^, the pseudogap^9–13^, and other broken-symmetry phases^14^ in carrier-doped Sr_2_IrO_4_.

The spectroscopic manifestation of the pseudogap in electron-doped Sr_2_IrO_4_ is analogous to that of the enigmatic pseudogap in the cuprates. Angle-resolved photoemission spectroscopy (ARPES) experiments on K-deposited or La-doped Sr_2_IrO_4_ revealed the antinodal pseudogap state with coherent nodal excitations upon electron doping^9, 10^. In K-deposited Sr_2_IrO_4_, the Fermi arc representing the pseudogap emerges at about 110 K for 0.7 monolayer coverage (~6% electron doping)^9^ and evolves into a nodal point with a *d*-wave symmetry at lower temperatures^8^. Scanning tunneling microscopy/spectroscopy (STM/STS) studies demonstrated that the nanoscale electronic phase separation occurred upon electron doping and the pseudogap states emerged around the dopant atoms^11, 12, 15^. Despite these observations, the origin of the pseudogap in this *J*_eff_ = 1/2 cuprate analog remains elusive. A roadblock to understanding this state is that its temperature evolution is not currently well defined. Although the ARPES data of K-deposited Sr_2_IrO_4_ suggested that the pseudogap developed at *T* ^*^ ≈ 110 K^9^, the temperature evolution of the pseudogap state is not included in the STS study on K-deposited Sr_2_IrO_4_^11^ and the ARPES/STS studies in (Sr_1−*x*_La_*x*_)_2_IrO_4_^10, 12^.

Infrared spectroscopy is well suited for investigating anomalous charge dynamics in correlated electron materials. Indeed infrared spectroscopy played critical roles in documenting the pseudogap phase in cuprates^16–21^, where for instance the electronic pseudogap inferred from nuclear magnetic resonance experiments was first identified in *c*-axis infrared conductivity data of YBa_2_Cu_3_O_6+*δ*_^16^. Therefore infrared spectroscopy experiments could shed valuable insights on the nature of the charge transport and the pseudogap phase in electron doped Sr_2_IrO_4_, potentially providing a useful comparator to cuprates as well. However, to the best of our knowledge, no infrared spectroscopic study in carrier-doped Sr_2_IrO_4_ has been reported before.

In this paper, we investigate the *ab*-plane (IrO_2_ plane) electronic response in (Sr_1−*x*_La_*x*_)_2_IrO_4_ (*x* = 0, 0.021, and 0.067) by using infrared spectroscopy. We observe that (Sr_1−*x*_La_*x*_)_2_IrO_4_ undergoes a filling-controlled insulator-to-metal transition with La doping. Quantitative analyses on the evolution of the charge dynamics of (Sr_1−*x*_La_*x*_)_2_IrO_4_ show that the change in the optical excitation energy between the *J*_eff_ = 1/2 Hubbard bands is negligible while its intensity is suppressed, indicating the robustness of the electronic correlations in electron-doped Sr_2_IrO_4_. The far-infrared conductivity of (Sr_0.933_La_0.067_)_2_IrO_4_ reveals a hallmark of the pseudogap: a gradual depletion of the conductivity below a threshold frequency quantifying the magnitude of the pseudogap 2Δ_PG_ ≈ 17 meV. The evolution of the conductivity data associated with the formation of the pseudogap is quite similar to the phenomenology in the *c*-axis pseudogap of the underdoped cuprates^16, 22, 23^. At high temperatures, the spectral shape of the far-infrared conductivity in (Sr_0.933_La_0.067_)_2_IrO_4_ is nearly frequency independent, indicating a bad metallic nature. With decreasing temperature across 100 K, the conductivity below 20 meV is gradually depressed, revealing the threshold structure. The temperature at which the pseudogap develops *T* ^*^ ≈ 100 K coincides with the previously reported onset temperature of short-range antiferromagnetic order^15^. Our results suggest that the pseudogap in electron-doped Sr_2_IrO_4_ arises from strong electronic correlations and persistent short-range antiferromagnetism.

## Results and Discussion

**Figure 1:**
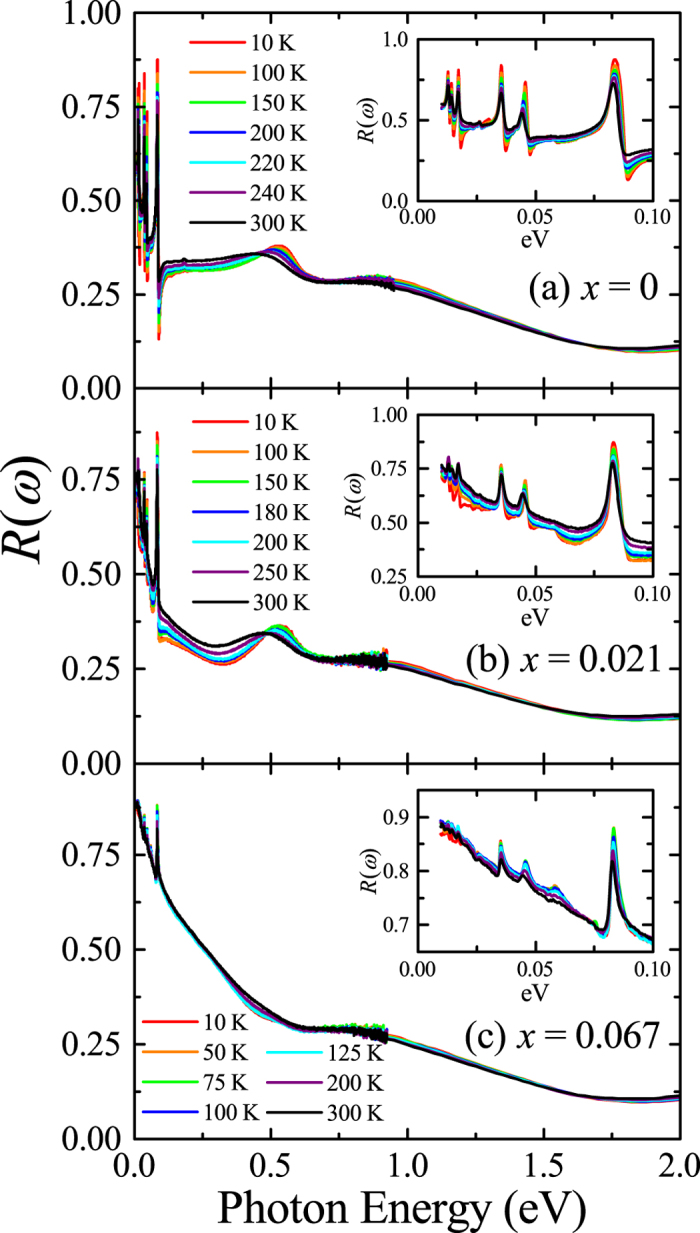
Temperature-dependent reflectivity spectra *R*(*ω*) of *ab*-plane of (**a**) Sr_2_IrO_4_ (*x = *0), (**b**) (Sr_0.979_La_0.021_)_2_IrO_4_ (*x = *0.021), and (**c**) (Sr_0.933_La_0.067_)_2_IrO_4_ (*x = *0.067). Insets: *R*(*ω*) below 0.1 eV.

Figure 1 shows the reflectivity spectra *R*(*ω*) of (Sr_1−*x*_La_*x*_)_2_IrO_4_ at various temperatures. The evolution of the low-energy *R*(*ω*) indicates an insulator-to-metal transition with electron doping. The far-infrared reflectivity spectra of Sr_2_IrO_4_, shown in the inset of Fig. 1(a), are dominated by sharp spikes due to infrared-active phonon modes. Two humps located at about 0.5 and 1 eV correspond to the optical excitations between the *J*_eff_ bands^1, 24, 25^. Upon electron doping, *R*(*ω*) below 0.1 eV acquires a spectral shape that rises toward lower frequency, indicating an electronic conduction. While the two humps at about 0.5 and 1 eV persist in (Sr_0.979_La_0.021_)_2_IrO_4_ [Fig. 1(b)], the lower-energy one appears to be suppressed in (Sr_0.933_La_0.067_)_2_IrO_4_ [Fig. 1(c)]. For (Sr_0.979_La_0.021_)_2_IrO_4_, the far-infrared *R*(*ω*) decreases continuously with decreasing temperature [inset of Fig. 1(b)], signaling an incoherent nature of the charge transport. The low-energy *R*(*ω*) of (Sr_0.933_La_0.067_)_2_IrO_4_ displays an anomalous temperature dependence [inset of Fig. 1(c)]. As the temperature decreases from 300 K to 100 K, the magnitude of *R*(*ω*) increases, which indicates an enhancement of the response from the itinerant carriers. However, as the temperature is lowered below 100 K, *R*(*ω*) below about 20 meV is suppressed. This feature is associated with the formation of a pseudogap.

**Figure 2:**
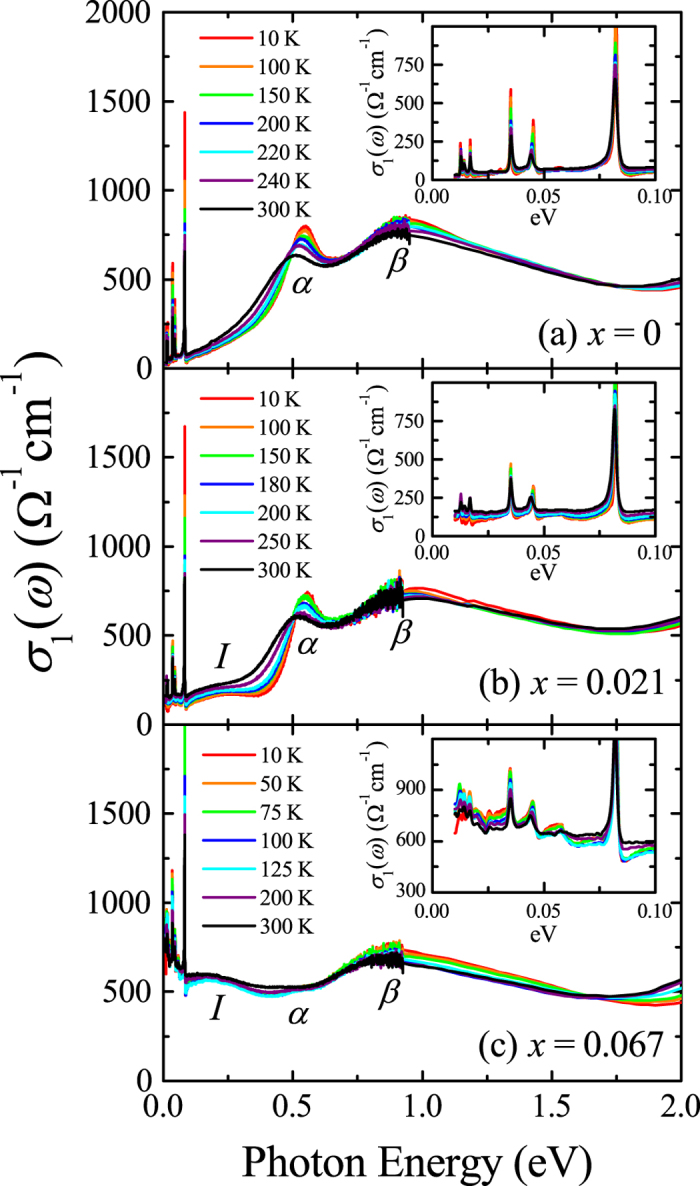
Real part of the optical conductivity spectra *σ*_1_(*ω*) of *ab*-plane of (**a**) Sr_2_IrO_4_ (*x = *0), (**b**) (Sr_0.979_La_0.021_)_2_IrO_4_ (*x = *0.021), and (**c**) (Sr_0.933_La_0.067_)_2_IrO_4_ (*x = *0.067). Insets: *σ*_1_(*ω*) below 0.1 eV.

Figure 2 displays the real part of the optical conductivity *σ*_1_(*ω*) spectra of (Sr_1−*x*_La_*x*_)_2_IrO_4_. The optical conductivity of Sr_2_IrO_4_ shows the characteristic two-peak structure of the *J*_eff_ = 1/2 Mott insulator. The degeneracy of *d* orbitals of the iridium ions is broken by the cooperation of crystal field and spin-orbit coupling forming *J*_eff_ states. The on-site Coulomb repulsion *U* splits the *J*_eff_ = 1/2 band into a lower Hubbard band (LHB) and an upper Hubbard band (UHB). Two peaks at about 0.5 eV and 1 eV labeled as *α* and *β* correspond to the transitions from the LHB to UHB and from the *J*_eff_ = 3/2 bands to the *J*_eff_ = 1/2 UHB, respectively^1, 24, 25^.

Electron doping induces the appearance of spectral weight (SW) below the Mott gap. The conductivity data of (Sr_0.979_La_0.021_)_2_IrO_4_ in Fig. 2(b) show a weak ingap excitation (peak *I*) centered at about 0.2 eV. It is interesting to note that the *α* and *β* peaks are robust against 4.2% electron doping. An increase in the doping concentration up to 13.4% electrons/Ir (*x* = 0.067) causes a significant enhancement of the ingap excitation and a concomitant suppression of the peak *α* [Fig. 2(c)], in contrast to the barely altered peak *β*. In the energy region below 0.1 eV, *σ*_1_(*ω*) at high temperature exhibits a weak Drude-like response, where the conductivity of (Sr_0.933_La_0.067_)_2_IrO_4_ below 0.1 eV rises slightly toward lower frequency. The evolution of this electronic response upon doping will be discussed later in detail.

**Figure 3:**
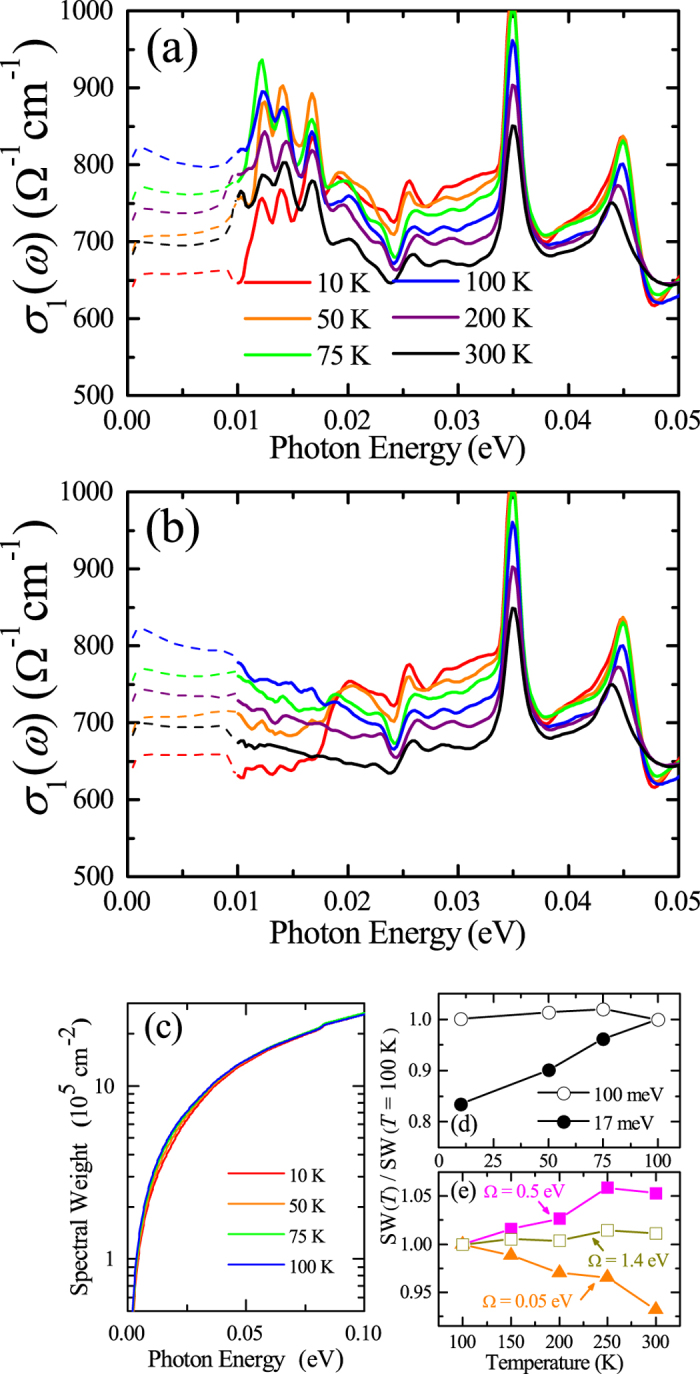
(**a**) The raw *ab*-plane *σ*_1_(*ω*) of (Sr_0.933_La_0.067_)_2_IrO_4_ in the energy region below 0.05 eV. In (**b**), we display the optical conductivity obtained by subtracting the contribution of the external phonon modes from the raw *σ*_1_(*ω*). The dashed lines represent the extrapolated *σ*_1_(*ω*) calculated from the Kramers-Kronig analysis of *R*(*ω*) with the Hagen-Rubens relation. (**c**) Frequency-dependent spectral weight SW(*ω*). (**d**) SW(17 meV) (solid circles) and SW(100 meV) (open circles) normalized to their corresponding SW’s at 100 K. (**e**) Temperature dependence of the spectral weight for cutoff Ω = 0.05 eV (solid triangles), 0.5 eV (solid squares), and *ω*_p_ ~ 1.4 eV (open squares).

A closer inspection of the low-energy *σ*_1_(*ω*) of (Sr_0.933_La_0.067_)_2_IrO_4_ reveals an infrared hallmark of the pseudogap, i.e., a gradual depletion of the conductivity with decreasing temperature^16, 22, 23^. In general terms, the pseudogap means a depletion of density of states^26, 27^. Thus the formation of the pseudogap implies the suppression of the optical conductivity below the energy of the pseudogap 2Δ_PG_. In Fig. 3(a), the optical conductivity below 50 meV is shown to highlight the pseudogap formation in (Sr_0.933_La_0.067_)_2_IrO_4_. The conductivity below the cutoff frequency of our experiments (dashed lines) were obtained by the Kramers-Kronig analysis of *R*(*ω*) with Hagen-Rubens low-energy extrapolation^28^ [See the Method section for details]. We stress that different extrapolation methods produce negligible variation in *σ*_1_(*ω*) in the energy region covered in our experiments. Although the presence of the phonon modes complicates the correct identification of the spectral shape, the formation of the pseudogap is evident. The magnitude of *σ*_1_(*ω*) below 50 meV increases with decreasing temperature from 300 K to 100 K. With further cooling below 100 K, the magnitude of *σ*_1_(*ω*) above 20 meV keeps increasing, in stark contrast to the suppression of *σ*_1_(*ω*) below 20 meV.

The formation of the pseudogap is better manifested after subtraction of the phonon contribution from the conductivity spectra [Fig. 3(b)]. Sr_2_IrO_4_ adopts the K_2_NiF_4_-type structure with *I*4_1_/acd space group^15^. Group theory predicts six infrared-active phonon modes^29, 30^, which is consistent with the number of observed phonon modes in our *σ*_1_(*ω*) [insets of Fig. 2]. The highest-energy mode at 82 meV corresponds to the stretching mode of the Ir-O bond. The other five phonon modes exist below 50 meV. Three external modes below 20 meV are related to the vibrations of the Sr ions against IrO_6_ octahedra and the remaining two bending modes at 35 and 45 meV are related to the modulation of the Ir-O-Ir bond angle^25, 29, 30^. The Drude-Lorentz oscillator model is employed to analyze the optical conductivity^28, 31^. The external phonon modes were fitted by three Lorentz oscillators. The electronic contribution to *σ*_1_(*ω*) at 300, 200, and 100 K (75, 50, and 10 K) was simulated by a broad Drude oscillator (a Drude and two Lorentz oscillators). Then the contribution from the external modes was subtracted from *σ*_1_(*ω*) to emphasize the sole electronic component to the conductivity in the low energy region [Fig. 3(b)]. Although the spectra are noisy, the phonon peaks can be isolated based on the comparison with *σ*_1_(*ω*) of Sr_2_IrO_4_ and (Sr_0.979_La_0.021_)_2_IrO_4_ where the phonon modes are distinct [insets of Fig. 2(a) and (b)]. At 300 K, the optical conductivity spectrum below 0.05 eV is almost flat other than a slight rise toward lower frequencies, suggesting the incoherent character of the charge transport. As the temperature decreases to 100 K, the magnitude of *σ*_1_(*ω*) increases continuously. In contrast, with the further lowering of temperature below 100 K, *σ*_1_(*ω*) below 20 meV is suppressed, which is consistent with the behavior of the resistivity data^15^. The conductivity data at 50 and 10 K display a clear gap region below about 17 meV. It should be noted that *σ*_1_(*ω*) above 20 meV is enhanced along with the pseudogap formation. This observation signals a transfer of the SW into higher energies. The temperature evolution of *σ*_1_(*ω*) indicates that the magnitude of the pseudogap 2Δ_PG_ is about 17 meV and the temperature *T* ^*^ below which the pseudogap develops is about 100 K.

It is interesting to note that the pseudogap behavior discovered in the *ab*-plane response of electron-doped Sr_2_IrO_4_ is similar to that of the *c*-axis pseudogap of underdoped YBa_2_Cu_3_O_6+δ_ (YBCO)^16, 17^. The *c*-axis conductivity of the underdoped cuprates is characterized by a featureless incoherent far-infrared response. The pseudogap was manifested by a gradual depletion of the *c*-axis conductivity below certain energy with decreasing temperature. Such change leads to the development of a threshold structure at 2Δ_PG_^16, 17^. The SW removed from the energy region below the pseudogap is shifted to higher energies^22, 32, 33^. Conversely, the signature of the pseudogap in the cuprates cannot be clearly identified from the in-plane optical conductivity due to the dominance of the coherent Drude-like peak but is represented by a drop in the frequency-dependent scattering rate below 2Δ_PG_^23^. The bad metallic character of the *ab*-plane response of (Sr_0.933_La_0.067_)_2_IrO_4_ may enable the identification of the pseudogap behavior from our conductivity data.

Aside from the similarities, a quantitative difference exists in the energy scale of the SW transfer due to the formation of the pseudogap in (Sr_1−*x*_La_*x*_)_2_IrO_4_ and the cuprates. In the underdoped cuprates, the energy scale of the SW transfer significantly exceeds the magnitude of the pseudogap^22, 32, 33^. In contrast, the conductivity data of (Sr_1−*x*_La_*x*_)_2_IrO_4_ clearly demonstrate that the SW is only shifted to the region close to the pseudogap energy; *σ*_1_(*ω*) right above 2Δ_PG_ is enhanced with the opening of the pseudogap. In order to estimate the energy scale of the redistribution of the SW due to the formation of the pseudogap, we analyzed the temperature dependence of the $SW(\omega )={\int }_{0}^{\omega }{\sigma }_{1}(\omega \text{'})d\omega \text{'}$ in the temperature region below *T* ^*^ ≈ 100 K. As shown in Fig. 3(c), the SW(*ω*) at *ω* < 2Δ_PG_ decreases upon entering the pseudogap phase. At *ω* > 2Δ_PG_, the SW(*ω*) at lower temperatures increases more rapidly with increasing energy which indicates the accumulation of the SW in the corresponding energy region. In Fig. 3(d), the temperature dependence of the SW(17 meV) and SW(100 meV) normalized to the corresponding 100 K SW’s are shown, from which we find that the formation of the pseudogap removes about 17% of the spectral weight below 2Δ_PG_ ≈ 17 meV and the sum rule is satisfied up to about 100 meV.

It is worthwhile comparing the infrared pseudogap to those revealed by other experimental techniques. An ARPES measurement on (Sr_0.95_La_0.05_)_2_IrO_4_ reported that the magnitude of the pseudogap at the antinodal point was 2Δ_PG_ ≈ 50 meV^10^. A STM experiment on (Sr_0.945_La_0.055_)_2_IrO_4_ suggested 2Δ_PG_ ≈ 70–300 meV^12^. These scales of the pseudogap are larger than the estimation from our infrared data. This may be attributed to the difference of the doping level. The La doping concentration of our sample is 6.7% which is larger than that of the crystals measured by the ARPES^10^ and STM^12^ studies. An ARPES measurement on surface-electron-doped Sr_2_IrO_4_ showed that the magnitude of the pseudogap decreases with increasing doping concentrations^9^. As the surface coverage increases from 0.5 monolayer to 0.7 monolayer which corresponds to the increase of electron doping concentration by about 8% in K-deposited Sr_2_IrO_4_, the magnitude of the pseudogap decreases from 80 meV to 20 meV. Assuming the linear relationship between the electron doping and the magnitude of the pseudogap, the ~8 meV decrease in the magnitude of the pseudogap per 1% extra electron doping should be respected. The electron doping concentration of our sample is 2*x = *13.4% electrons/Ir which is 3.4% larger than 2*x* = 10% of the sample used in the ARPES study^10^. Therefore, the magnitude of the pseudogap of our sample is expected to be about 30 meV smaller than that of ~50 meV from the ARPES study, resulting in a magnitude of about 20 meV which is consistent with our observation.

The temperature *T* ^*^ ≈ 100 K at which the pseudogap develops in our data provides a clue on the origin of the pseudogap. Recent magnetization and neutron scattering experiments in (Sr_1−*x*_La_*x*_)_2_IrO_4_ revealed that while long-range antiferromagnetic order collapses beyond *x* = 0.02, short-range order survives up to the highest doping concentrations^15^. The onset temperature of the short-range antiferromagnetic order *T*_SRO_ in (Sr_0.94_La_0.06_)_2_IrO_4_ is about 125 K and further decreases with increasing *x*. Thus, the *T*_SRO_ in (Sr_0.933_La_0.067_)_2_IrO_4_ is expected to be in the vicinity of 100 K where the pseudogap emerges. This correlation suggests that the short-range antiferromagnetic order may be responsible for the infrared pseudogap.

**Figure 4:**
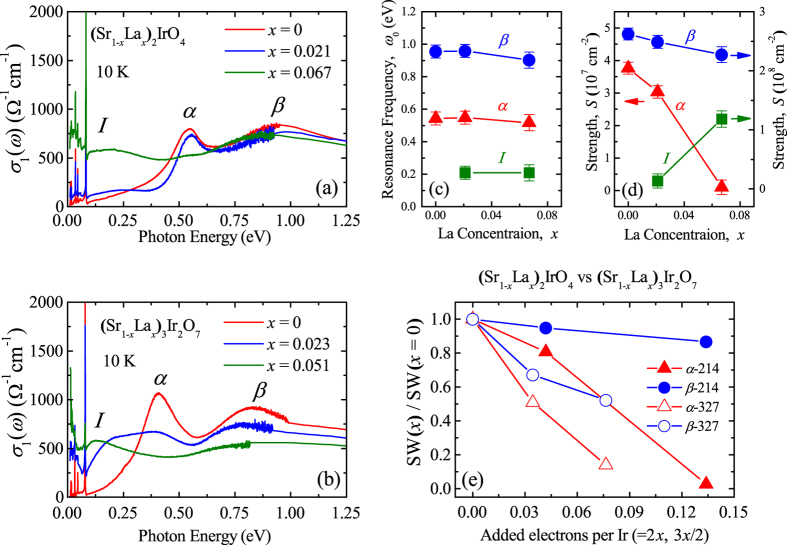
*σ*_1_(*ω*) of (**a**) (Sr_1−*x*_La_*x*_)_2_IrO_4_ and (**b**) (Sr_1−*x*_La_*x*_)_3_Ir_2_O_7_ at 10 K. (**c**) Resonant frequencies of the peaks *I* (solid squares), *α* (solid triangles), and *β* (solid circles). (**d**) Strengths of the peaks *I*, *α*, and *β*. (**e**) Normalized spectral weights of the peaks *α* (red triangles) and *β* (blue circles) SW(*x*)/SW(*x = *0) of (Sr_1−*x*_La_*x*_)_2_IrO_4_ (solid symbols) and (Sr_1−*x*_La_*x*_)_3_Ir_2_O_7_ (open symbols).

In order to gain more insights on the pseudogap as well as the electronic response in the (Sr_1−*x*_La_*x*_)_2_IrO_4_ system, we now elaborate on the doping evolution of *σ*_1_(*ω*) at 10 K [Fig. 4(a)]. The doping-induced changes in *σ*_1_(*ω*) are limited in the energy region below 0.6 eV where the excitation between the *J*_eff_ = 1/2 bands is the main contributor. We analyzed *σ*_1_(*ω*) by using the Drude-Lorentz oscillator model^28, 31^:

1 $${\sigma }_{1}(\omega )=\frac{1}{4\pi }\frac{{S}_{D}{\gamma }_{D}}{{\omega }^{2}+{{\gamma }_{D}}^{2}}+\frac{1}{4\pi }\sum _{j}\frac{{S}_{j}{\gamma }_{j}{\omega }^{2}}{{({{\omega }_{j}}^{2}-{\omega }^{2})}^{2}+{{\gamma }_{j}}^{2}{\omega }^{2}}.$$

The first and second terms represent the Drude and the Lorentz oscillators, respectively. *S*_*D*_ and *γ*_*D*_ (*S*_*j*_ and *γ*_*j*_) denote the strength and the scattering rate of the Drude (Lorentz) oscillator, respectively. *ω*_*j*_ in the second term denotes the resonance frequency of the Lorentz oscillator. The strength of the oscillator is proportional to its SW. Figure 4(c–e) show the results of the Drude-Lorentz oscillator analysis. We first note that the positions of the peaks *α* and *β* do not change evidently with *x* in (Sr_1−*x*_La_*x*_)_2_IrO_4_ [Fig. 4(c)]. On the other hand, the SW’s of the optical excitations exhibit a significant doping evolution. At *x* = 0.021, the SW’s of the peaks *α* and *β* slightly diminish and a weak ingap excitation appears [Fig. 4(d)]. As *x* increases to 0.067, the peak *α* is drastically suppressed and the peak *I* acquires the SW, whereas the SW of the peak *β* is almost untouched.

The negligible shift of the peaks *α* and *β* revealed in our analysis suggests that the magnitude of the electronic correlations does not vary appreciably with electron doping, which has an implication on recent discussions on the role of the electronic correlations in La-doped Sr_2_IrO_4_. An ARPES experiment on (Sr_1−*x*_La_*x*_)_2_IrO_4_ reported that the Mott gap closed due to the merging between the *J*_eff_ = 1/2 LHB and UHB with electron doping^10^. This observation was attributed to the reduction of the on-site Coulomb repulsion *U*. However, the first-principles calculation study pointed out that *U* did not change noticeably at low dopings and could only be fully suppressed upon 80% electron doping^34^. Another ARPES study showed that the *J*_eff_ = 1/2 bands were consistently separated upon electron doping. It was also observed that the photoemission intensity due to the *J*_eff_ = 1/2 LHB was suppressed significantly and the SW was shifted into the gap while the intensity from the *J*_eff_ = 3/2 bands did not change evidently^35^. Our data agree with the latter ARPES result and suggest that the electronic correlations remain robust against electron doping in the (Sr_1−*x*_La_*x*_)_2_IrO_4_ system. An observation of the phase separated state in (Sr_1−*x*_La_*x*_)_2_IrO_4_ also supports the persistence of the electronic correlations^12^.

Dynamical mean-field theory study of the optical responses in several cuprates suggested that the degree of the electronic correlations can be estimated from the temperature dependence of the low-energy spectral weight^36, 37^. Toschi *et al*. found that the spectral weight followed a quadratic temperature dependence

2 $$SW({\rm{\Omega }},T)=SW({\rm{\Omega }},0)-B({\rm{\Omega }}){T}^{2},$$

for any infrared cutoff below which intraband responses are main contributors to *σ*_1_(*ω*)^36, 37^. The electronic correlations were manifested in an unexpectedly large magnitude of the coefficient *B*(Ω = *ω*_p_) where *ω*_p_ is the plasma frequency. The plasma frequency of (Sr_0.933_La_0.067_)_2_IrO_4_ obtained from the fit of *σ*_1_(*ω*) is about 1.4 eV where the contribution from the interband transition, the peak *β*, is significant. Therefore one cannot apply the equation (2) with Ω = *ω*_p_ to (Sr_0.933_La_0.067_)_2_IrO_4_ for assessing the degree of the electronic correlations. The SW(Ω = 0.05 eV) decreases with increasing the temperature. However, the quadratic temperature dependence is not evident. We can observe a signature of the electronic correlations in the SW(Ω = 0.5 eV, *T*) data which includes the contribution from the intraband responses, i.e., the Drude-like mode and the ingap excitaiton. We find that the SW(Ω = 0.5 eV, *T*) increases at higher temperatures [Fig. 3(e)], which indicates the incoherent nature of the low-energy electronic response of (Sr_0.933_La_0.067_)_2_IrO_4_ possibly due to the electronic correlations^38^.

A direct comparison between *σ*_1_(*ω*) of the single-layer (Sr_1−*x*_La_*x*_)_2_IrO_4_ and the bilayer (Sr_1−*x*_La_*x*_)_3_Ir_2_O_7_ systems further highlights the importance of the electronic correlations in the charge dynamics of the single-layer system. Figures 4(a) and (b) illustrate that (Sr_1−*x*_La_*x*_)_2_IrO_4_ has a stronger propensity for localizing the doped charge carriers. Although less electrons are doped in (Sr_0.949_La_0.051_)_3_Ir_2_O_7_ (7.65% electrons/Ir doping) than in (Sr_0.933_La_0.067_)_2_IrO_4_ (13.4% electrons/Ir doping), a coherent Drude-like peak without any trace of the pseudogap is registered in the bilayer compound^39^. The evolutions of the SW’s of the peaks *α* and *β* also support the notion of the stronger tendency for charge localization in the single-layer system. Figure 4(e) displays the changes in the SW’s of the peaks *α* and *β* of the two systems which are normalized to those of their corresponding parent compounds. The SW’s of the peaks *α* and *β* in (Sr_0.977_La_0.023_)_3_Ir_2_O_7_ (3.45% electrons/Ir doping) decrease by about 50% and 33%, respectively. On the other hand, the SW’s of the two peaks in (Sr_1−*x*_La_*x*_)_2_IrO_4_ decrease substantially slowly. The SW’s of the peaks *α* and *β* in (Sr_0.979_La_0.021_)_2_IrO_4_ (4.2% electrons/Ir doping) are suppressed by about 19% and 5% respectively, suggesting the strong influence of the electronic correlations for inhibiting the delocalization of the doped electrons.

The distinct behavior of *σ*_1_(*ω*) in (Sr_1−*x*_La_*x*_)_2_IrO_4_ compared to that of (Sr_1−*x*_La_*x*_)_3_Ir_2_O_7_ may be attributed to the weaker mixing between the spin-orbit split *J*_eff_ = 1/2 and 3/2 in the former system. In the Ruddlesden-Popper series iridates Sr_*n*+1_Ir_*n*_O_3*n*+1_, the dimension determined by *n* can control the effective bandwidth of the *J*_eff_ bands. The increase in the dimensionality enhances the interlayer coupling and the mixing between the *J*_eff_ = 1/2 and 3/2 bands^40^. Thus, the effective bandwidth of the *J*_eff_ bands scale with *n*. The current observations indicate that only the *J*_eff_ = 1/2 bands are relevant for the low-energy charge dynamics in (Sr_1−*x*_La_*x*_)_2_IrO_4_. This suggests that the mixing is negligible and the magnitude of the effective electronic correlations defined by the ratio of *U* to the bandwidth *W* is the strongest in the single-layer system. The suppressions of the peaks *α* and *β* with electron doping in the bilayer system can be naturally explained in terms of the increase in the mixture and the effective bandwidth of the *J*_eff_ bands with increasing *n*.

## Conclusion

The electronic response of (Sr_1−*x*_La_*x*_)_2_IrO_4_ (*x* = 0, 0.021, and 0.067) upon electron doping is explored by infrared spectroscopy. The conductivity data show that the energy value of the Mott-gap excitation, i.e., the optical transition between the *J*_eff_ = 1/2 Hubbard bands remains almost untouched across the filling-controlled insulator-to-metal transition. It is established that only the optical excitation between the *J*_eff_ = 1/2 Hubbard bands was suppressed by electron doping, which is in sharp contrast to the behavior of *σ*_1_(*ω*) in (Sr_1−*x*_La_*x*_)_3_Ir_2_O_7_ where the optical excitation from the *J*_eff_ = 3/2 bands to the *J*_eff_ = 1/2 upper Hubbard band is also suppressed. In metallic (Sr_0.933_La_0.067_)_2_IrO_4_, the low-energy conductivity spectra are dominated by incoherent excitations. All these observations suggest the persistence of the strong electronic correlations upon electron doping in (Sr_1−*x*_La_*x*_)_2_IrO_4_. The signature of the pseudogap is identified in the conductivity data of the bad metallic compound (Sr_0.933_La_0.067_)_2_IrO_4_, where a gradual depletion of the far-infrared conductivity with decreasing the temperature below *T* ^*^ ≈ 100 K is observed. The magnitude of the infrared pseudogap 2Δ_PG_ at this concentration is found to be about 17 meV and the pseudogap temperature *T* ^*^ estimated from the data is in the vicinity of the onset temperature of the short-range antiferromagnetic order^15^. Our work indicates that the strong electronic correlations and remnant antiferromagnetism are responsible for the pseudogap in (Sr_1−*x*_La_*x*_)_2_IrO_4_.

## Methods

Single crystals of (Sr_1−*x*_La_*x*_)_2_IrO_4_ (*x* = 0, 0.021, and 0.067) were grown using flux technique. Stoichiometry and the La concentrations were checked by energy dispersive spectroscopy measurements. Details of the growth and the characterizations of the single crystals were described in ref. 15. We measured the *ab*-plane reflectivity spectra *R*(*ω*) in the energy region between 10 meV and 1 eV as a function of temperature by using a Fourier-transform infrared spectrometer (Bruker vertex 70 v). To compensate the effect of rough surfaces, we use *in-situ* gold overcoating technique^41^. We measured the temperature-dependent dielectric constants in the energy region between 1 and 5 eV by using spectroscopic ellipsometer (VASE, J. A. Woollam Co.). We obtained the complex optical conductivity by the Kramers-Kronig analysis^28^. For the extrapolation of *R*(*ω*) below 10 meV for insulating Sr_2_IrO_4_, constant values that match with the magnitude of *R*(*ω* = 10 meV) were chosen^28^. The Hagen-Rubens relation^28^ was used for the low-energy extrapolation of *R*(*ω*) for (Sr_0.979_La_0.021_)_2_IrO_4_ and (Sr_0.933_La_0.067_)_2_IrO_4_. We verified that the conductivity data at *ω* ≥ 10 meV did not depend on the extrapolation methods.
